# Effect of Melatonin on the Expression of Apoptotic Genes in Vitrified-thawed Spermatogonia Stem Cells Type A of 6-Day-Old Mice 

**Published:** 2013-08

**Authors:** Mohammadreza Gholami, Ghasem Saki, Masoud Hemadi, Ali Khodadadi, Javad Mohamma-di-asl

**Affiliations:** 1 Department of Anatomy, Lorestan University of Medical Sciences, Khoramabad, Iran; 2 Physiology Research Center, Ahvaz Jundishapur University of Medical Sciences (AJUMS), Ahvaz, Iran; 3 Fertility, Infertility and Perinatology Research Center, Ahvaz Jundishapur University of Medical Sciences (AJUMS), Ahvaz, Iran; 4 Department of Immunology, Ahvaz Jundishapur University of Medical Sciences (AJUMS), Ahvaz, Iran; 5 Departmentof Medical Genetics, Ahvaz Jundishapur University of Medical Sciences(AJUMS), Ahvaz, Iran

**Keywords:** Apoptosis, Melatonin, Testes, Vitrification

## Abstract

***Objective(s):*** Being secreted by the pineal gland, melatonin induces cell proliferation in normal cells and induced apoptosis in cancer cells. The purpose of this study was to investigate effects of melatonin on main components and the expression of apoptotic genes in vitrified-thawed testicular germ cells of 6- day–old mice.

***Materials and Methods:*** Testes of neonate Balb/c mice were vitrified- thawed under standard condition with or without the addition of 100 μM melatonin to both vitrification and thawing solutions. Subsequently, Vitrified-*thawed whole* testes were digested under standard condition and spermatogonial stem cells type A were separate in the suspension with CD90.1 (Thy1.1^+^) micro beads. Extraction of RNA and synthesis of cDNA was performed. Expression levels of apoptotic genes (Fas, P53, BCL-2 and BAX) were determined using Real-time PCR.

***Results*** *:* With all genes being expressed, level of expression for Fas was higher and for that of P-53 was lower than the remaining genes.

***Conclusion*** *:* Melatonin may cause apoptosis in cells being damaged under the influence of freezing thawing process. In order to examine the exact effects of melatonin on spermatogonia stem cells apoptosis, additional studies are required.

## Introduction

Spermatogenesis is a phenomenon at the beginning of puberty in which the sperm is resulted from mitotic and meiotic divisions of spermatogonia stem cells (1(. In cancers, the mentioned process is naturally disrupted in patients being treated with radiotherapy and chemotherapy, which may lead to infertility after treatment. It should be noted that about 2% of malignant cancers occur in children and toddlers (age less than 14 years old). In America, 8600 children are diagnosed with cancer every year. Radiotherapy and chemotherapy for treatment of childhood cancers such as lymphoma, neuroblastoma, Hodgkin's lymphoma, osteoarthritis sarcoma, sarcoma Ewins's, rhabdomyosarcom, Wilms' tumor and non-Hodgkin lymphoma, lead to impaired testicular activity during adolescence )^2^(. Compared with utilization of the testis and spermatogonia stem cells, cryopreservation of sperm and embryos is an effective technique. Seeking a solution for infertility in cancer patients, several attempts have been made. Nowadays, large amounts of semen have been frozen and the fertility rate is much improved, especially with ICSI  (intracytoplasmic sperm injection)technique (3, 4). However, these techniques are only for adult patients, who have the ability to produce sperm and have a partner. Spermatogonia stem cells are more resistant to damages caused by the freezing-thawing process compared to mature sperms. Spermatogonia stem cells are relatively inactive and lack acrosome (5-7). 

Due to the large size of spermatogonia stem cells, permeability antifreezing agent is easier and fewer cytogenetic abnormalities occur during freezing (7). However, *in vitro* culture of spermatogonia stem cells is difficult compared with mature sperms (7, 8). 

Nowadays, researchers have turned their attention to spermatogonia stem cells transplantation. Spermatogonia stem cells are applied for transplantation using techniques such as frozen-thawed or fresh separately or linked to other testes. This techniques have been introduced as proper methods that can be held accountable for improving and sustaining the next generation in the cancer diseases. Recently, several researches have been done to optimize the Cryopreservation media. Cryopreservation is a procedure to preserve fertility after treatment with chemotherapy agents or radiotherapy (9-11). Immature testicular tissue harvested from the testis are immersed in cryoprotectant media and transferred immediately to liquid nitrogen (rapid-freezing) or refrigerator to decrease its temperature which is the base of programs (slow-freezing) (12-14). Rapid-freezing or vitrification, is a better strategy as it prevents ice crystal formation and biologically damaging effects (15). Nevertheless, vitrification and thawing induce damage to cells including reduced viability, induction of apoptosis, loss of integrity of DNA, breakdown of cell membrane, formation of oxygen free radicals, solution effects and intracellular ice crystals (15-20). Therefore, reducing the injury to cells in the process of vitrification and thawing is considered necessary. Apoptosis or programmed cell death is a very important functional and physiological phenomenon that is necessary for cells proliferation and differentiation (21). Apoptosis of germ cells is visible under physiological and pathological conditions. Manifestations of this phenomenon are crumpled nucleus and cytoplasm, broken membrane and formation of apoptotic bodies (21). With cells being exposed to stresses such as oxidative stress, chemicals and radiation, apoptosis is enhanced (21). We had previously shown that supplementation of vitrified-thawed media with melatonin do not protect spermatogonia stem cells against cryopreserved-induced injury (22). In the present study, effect of melatonin on apoptosis key components and genes is examined. Melatonin is a small biological molecule that is secreted in the pineal gland and other organs e.g. retina, testis (23-27). Effects of melatonin are studied on many regulatory functions of cells such as immune response, cell signaling, protection of fatty acids from oxidation and nuclear DNA from damage, control over tumor growth and inhibition of cell proliferation, oncostatic action, antiapoptotic effect on many normal cells, enhancement or promotion of apoptosis in the tumor cells and significant anti-aging properties (23-27). Anti-apoptotic effects of melatonin on normal cells are demonstrated as induction of cell cycle blockage and apoptosis in tumor cells during cell division (26, 28-32). In this study, effect of melatonin on expression of apoptotic genes in vitrified-thawed testicular germ cells of 6-day old mouse was investigated.

## Materials and Methods

All experiments were performed in accordance with principles of laboratory animal care. Male 6 -day old BALB/c mouse pups (N=80) were obtained from physiology research center. Mice were euthanized by excessive doses of ketamine HCl (80 mg/kg) and xylazine (10 mg/kg) (Pharmacia and Upiohn, Erlangen, Germany) (33) in accordance with the protocols approved by the Ahvaz Jundishapur University Medical Science Animal Care and Use Committee. Every effort was made to minimize the number of animals used and their suffering. Male mice were randomly assigned to *one* of the two experimental *groups*. After collecting the intact testes from the mice, they were vitrified in the vitrification medium that was supplemented with melatonin (group A) or a medium without melatonin (group B).

***Vitrification procedure***

Testes were transferred to vitrification solution 1 (V.S. 1) that contained 0.5 molar sucrose, 7.5% ethylene glycol, 7.5% DMSO and 100 μM melatonin. After 10 min, testes were transferred to vitrification solution 2 (V.S. 2), containing 0.5 molar sucrose, 15% ethylene glycol, 15% DMSO and 100 μM melatonin for 10 min. Samples were subsequently transferred to V.S 3 that contained 0.5 molar sucrose, 15% ethylene glycol, 15% DMSO, 100 μM melatonin and 20% FBS. Samples were transferred to liquid nitrogen tank after a 10-min period.

***Thawing procedure***

After removal from liquid nitrogen, samples were maintained in the room temperature for 30 sec and were subsequently held in water bath 37°C until defreeze. They were then transferred to thawing solution 1 (T.S.1) in 4°C that contained 0.5 molar sucrose and 100 μM melatonin. After 5 min, samples were transferred to thawing solution 2 (T.S.2) in 4°C that contained 0.25 molar sucrose and 100 μM melatonin. Samples, after 5 min, were transferred to thawing solution 3 (T.S.3) in 4°C containing 0.125 molar sucrose and 100 μM melatonin.

***Digestion of 6-Day-Old mouse testes***

Cell digestion was conducted according to Milazzo *et al* with little change (34). Briefly, after removal of tunica albogina, 6-day Old mouse testes were digested in two steps. In the first step, 10 testes were incubated in 1mg/ml collagenase type ΙV and 200-700 µg/ml DNaseΙ for 15 min in 37°C with slow pipetting. After centrifugation in 100× g for 5 min, in the second steps, supernatant was discarded and cells were re-suspended in 1cc trypsin-EDTA (sigma) and 200 µg/ml DNaseΙ for 5 min in 37°C. Trypsin was inactivated with adding 10% FBS to the cell suspension.

***Separation with MACS***

CD90.1 (Thy1.1^+^) was used to detect spermatogonia stem cells type A. The procedure was performed according to the manufacturer’s instructions (Miltenyi Biotec, order no. 130-094-523). In the brief, 10^7^ total cells were centrifuged at 300×g for 10 min. Cell pellet re-suspend in 90 µl of buffer. Buffer solution contained phosphate-buffered saline (PBS), pH 7.2, 0.5% bovine serum albumin (BSA), and 2 mM EDTA by diluting MACS BSA stock solution (# 130-91-376) 1:20 in autoMACS rinsing solution (# 130-091-222). 10 µl of CD90.1 micro-beads was added, well mixed and incubated for 15 min in the refrigerator (2-8˚C). Cells were washed by adding 1-2 ml of buffer and centrifuged at 300×g for 10 min. Up to 10^8^ cells were re-suspend in 500 µl of buffer. Afterward, the cell suspension was loaded onto a MACS Column, which was placed in the magnetic field of a MACS Separator.

***RNA isolation***

Extraction of RNA was performed by RNeasy Mini Kit, according to manufacturer’s catalog (Qiagen, Cat. no. 74104). The purity and concentration of RNA was performed by measuring the absorbance at 260 nm (A_260_) using a spectrophotometer.

***cDNA synthesis***

cDNA synthesis was performed by the unique QuantiTect Reverse Transcription Kit, according to manufacturer’s catalog (Qiagen, Cat. no. 205311).

***Real-time PCR***

Real-time PCR was performed using Applied Bioscience 7500 fast with SYBR Green detection for gene expression analysis. Twenty seven reaction amplification cycles was performed. Each reaction cycle consisted of: 15 sec at 94˚C, 30 sec at 60˚C and 30 sec at 72˚C. Control mixture consisted of PCR mixture without cDNA. Primers list are shown in the Table 1.

## Results

***Real-time PCR***

**Figure 1:**
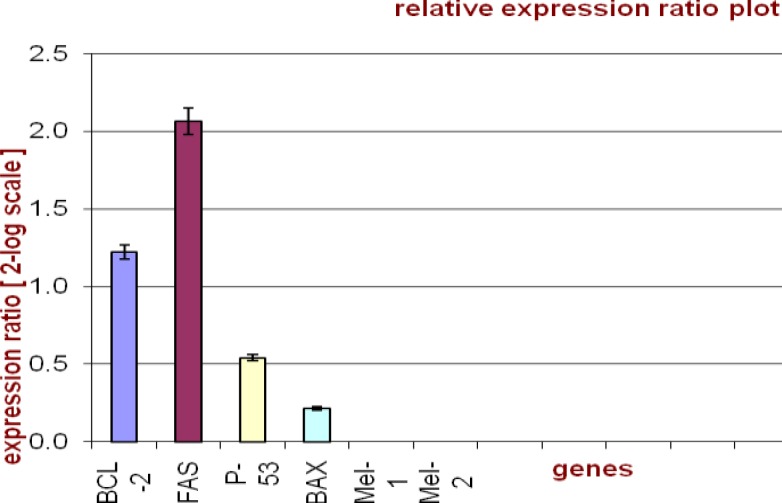
Expression of apoptotic genes. Mt1 and Mt2 were not expressed in spermatogonia stem cells. This graph was applied with rest-RG software.

Expression levels of apoptotic genes (Fas, P53, BCL-2 and BAX) were determined using Real-time PCR. The results were analyzed with LinRegPCR and Rest-RG softwares. Figure 1 show that all genes are expressed and Fas gene expression level is greater than the rest, while it is lower for P-53 comparing to other genes. Gene expressions of melatonin receptors (MT1, MT2) in type A spermatogonia stem cells were not observed.

## Discussion

 Apoptosis or programmed cell death is a very important functional and physiological phenomenon that is necessary for proliferation and differentiation of cells (21, 35). Germ cell apoptosis under physiological and pathological conditions can be observed. 

**Table 1:** primer feature used for Real-time PCR

Primer name	Primer sequence	Ref./GenBank
P53	F: GGAGTATTTGGACGACCG R: TCAGTCTGAGTCAGGCCC	NM011640
Bcl-2	F: TAAGCTGTCACAGAGGGGCT R: TGAAGAGTTCCTCCACCACC	NM009741
Bax	F: CGAGCTGATCAGAACCATCA R: GAAAAATGCCTTTCCCCTTC	NM007527
Fas	F: GAGAATTGCTGAAGACATGACAATCC R: GTAGTTTTCACTCCAGACATTGTCC	(46)
Mt1	F: TGCCACAGCCTCAAGTACGACA R: ACGCTGAGCTGACAGACTGGGT	NM_008639.2
Mt2	F:CCCATCTACATCAGCCTCGT R:ATTCGCAGGTAGCAGAAGGA	
GAPDH	F: GTGAAGGTCGGTGTGAACGG R: GATGCAGGGATGATGTTCTG	NM008084

Any disorder or cease in the physiological process of apoptosis leads to the formation of testicular cancer  (35). Review of key components and genes involved in the apoptosis pathway and the quality of induction of this phenomenon is necessary. Two main pathways exist for apoptosis including the internal and external routes (36). The internal or mitochondrial pathway includes activation of BAX/Bcl-2 with some pores in the membrane of the mitochondria, causing discharge of cytochrome C which will ultimately lead to apoptosis (37). In the external route, cell death receptors exist as family members of the TNF/NGF in the cell membrane (38). Several death receptors such as TNF, DR3, Fas and CD95 are present (38). Fas receptor exists in spermatogonia stem cells. The study results showed that melatonin enhanced expression of apoptotic genes such as BAX, Bcl-2, Fas and P53. Previous researches have shown that apoptosis induced by physiological and pathological process can be reduced by melatonin (23, 39). Melatonin has antioxidant effects (23, 40). Mechanisms involved in the anti-apoptotic property of melatonin are unknown (23). The anti-apoptotic effects through stimulating the receptor or its ability to remove free radicals are not still precisely known (23). In malignant cells, including human breast cancer cells MCF-7 category, addition of micromoles concentrations of melatonin increase apoptosis (41). In MCF-7 cells line, melatonin increases the expression of P53 (41). This finding about MCF-7 cells line apoptosis is consistent with the results of this paper. However, melatonin has anti-apoptotic effects in U937 cells, a category of human monocytes (29) which is inconsistent with the results of this article. Some investigators suggest that melatonin can be used as an antioxidant to prevent apoptosis in neurons (42). Melatonin, via receptor-dependent or receptor-independent mechanisms, can prevent internal apoptosis in tumor cells (36). Indirectly, melatonin membrane receptor activation prevents phosphorylation of P38 and JNK that leads to prevention of BAX/Bcl-2 pathway activation (43, 44). On the other hand, in tumor cells, activation of apoptosis pathways are caused by mechanisms dependent on melatonin receptor and receptor-independent . In such conditions, melatonin decreases the antioxidant defense mechanisms (36). Thus, intracellular ROS production and printing can directly increase apoptosis and expression of the apoptotic proteins such as P38, JNK and P53 (36). This leads to the activation of apoptotic pathways of BAX/Bcl-2 (36). Melatonin binding to DNA can induce ROR  (retinoid Orphan Receptors) (36) which is a nuclear receptor for Melatonin. In addition, melatonin induces apoptosis in Ewing’s sarcoma cell line and increases the expression of Fas and Fas L (45). Such increase is responsible for the induction of cell death by indolamine. Melatonin also provides a temporary increase in intracellular oxidants and the nuclear factor-kappaB activation (36, 45). The above-mentioned outcomes are consistent with results obtained in this study.

## Conclusion

 In sum, it can be stated that melatonin may cause apoptosis in cells damaged in the process of freezingthawing. In order for more accurate evaluation of the effect of melatonin on the process of freezing thawing and to determine the exact mechanism on apoptosis in spermatogonia stem cells, further studies are suggested.
